# Supplementary material for: Novel Phenothiazine-Bridged Porphyrin-(Hetero)aryl dyads: Synthesis, Optical Properties, In Vitro Cytotoxicity and Staining of Human Ovarian Tumor Cell Lines
Source: Int J Mol Sci. 2020 Apr 30;21(9):3178. doi: 10.3390/ijms21093178 (PMC7246510; doi:10.3390/ijms21093178)
Supplement: Supplementary file 1 [file ijms-21-03178-s001.pdf]

Novel phenothiazine bridged porphyrin-(hetero)aryl dyads: synthesis, optical properties, *in vitro* cytotoxicity and staining of human ovarian tumor cell lines.

Eva Molnar [1], Emese Gal [1], Luiza Gaina [1], Castelia Cristea [1*], Luminita Silaghi-Dumitrescu [1], Eva Fischer-Fodor [2,3], Maria Perde-Schrepler [2], Patriciu Achimas-Cadariu [4,5], Monica Focsan[6]

Supplementary materials

Contents:

NMR spectra of *5,15-diphenyl-10,20-bis(7-bromo-10-methyl-10H-phenothiazin-3-yl)-21,23H-porphyrin* **3a (**figure S1 and S2)

NMR spectra of *5,10,15-triphenyl-20-(8-chloro-10-methyl-10H-phenothiazin-3-yl)-21,23-Zn-porphyrin* ***5a*** **(**figure S3 and S4)

NMR spectra of *5,15-diphenyl-15,20-bis(7-(3′-formyl-10′-methyl-10′H-phenothiazin-7yl)-10-methyl-10H-phenothiazin-3-yl)-21,23H-porphyrin* **13** (figures S5-S8)

HRMS spectrum of *5,15-diphenyl-10,20-bis(7-bromo-10-methyl-10H-phenothiazin-3-yl)-21,23H-porphyrin* **3a** (figure S9)

HRMS spectrum of *5,10,15-triphenyl-20-(7-bromo-10-methyl-10H-phenothiazin-3-yl)-21,23-Zn-porphyrin* **4a** (figure S10)

HRMS spectrum of *5,10,15-triphenyl-20-(8-chloro-10-methyl-10H-phenothiazin-3-yl)-21,23-Pd-porphyrin* ***5b*** (figure S11)

HRMS spectrum of *5,10,15-triphenyl-20-(8-chloro-10-methyl-10H-phenothiazin-3-yl)-21,23-Cu-porphyrin* **5d**

(figure S12)

HRMS spectrum of *5,15-diphenyl-10,20-bis(7-bromo-10-methyl-10H-phenothiazin-3-yl)-21,23-Cu-porphyrin* **6d**

(figure S13)

Correlations between fluorescence intensity and concentration for 9, 13, 15 fluorescent dyes against ovarian adenocarcinoma A2780cis and OVCAR3 cells (figure 14-15)

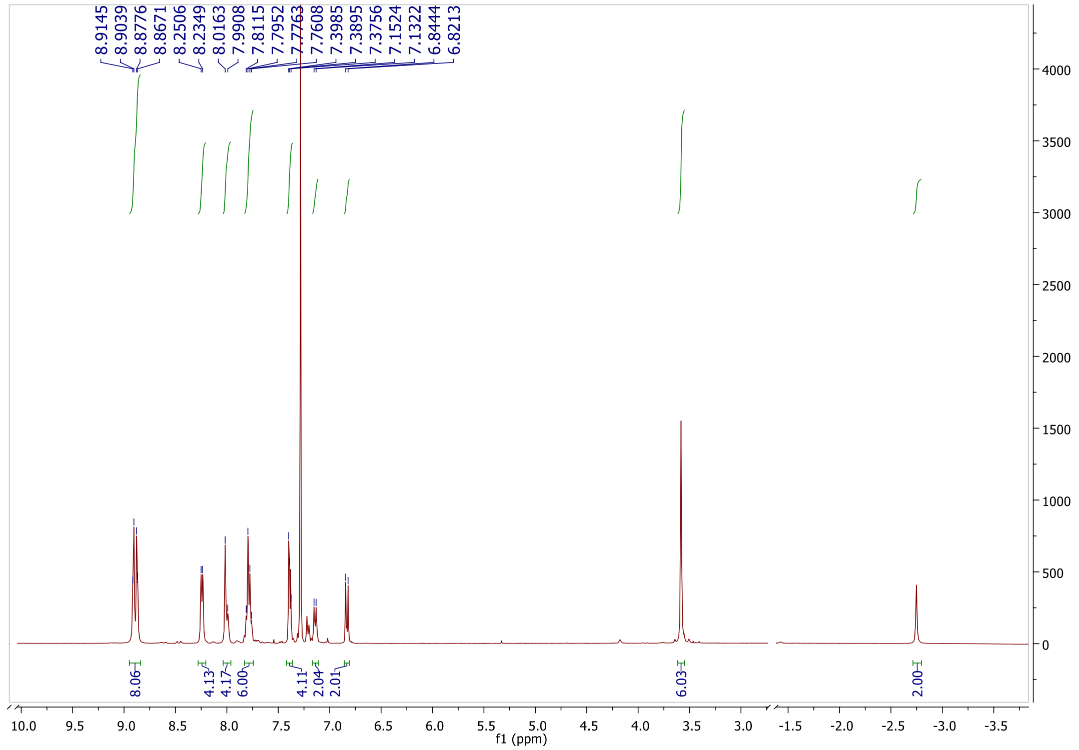

Figure S1. ^{1}H-NMR 400 MHz spectrum of **3a** ($CDCl_3$)

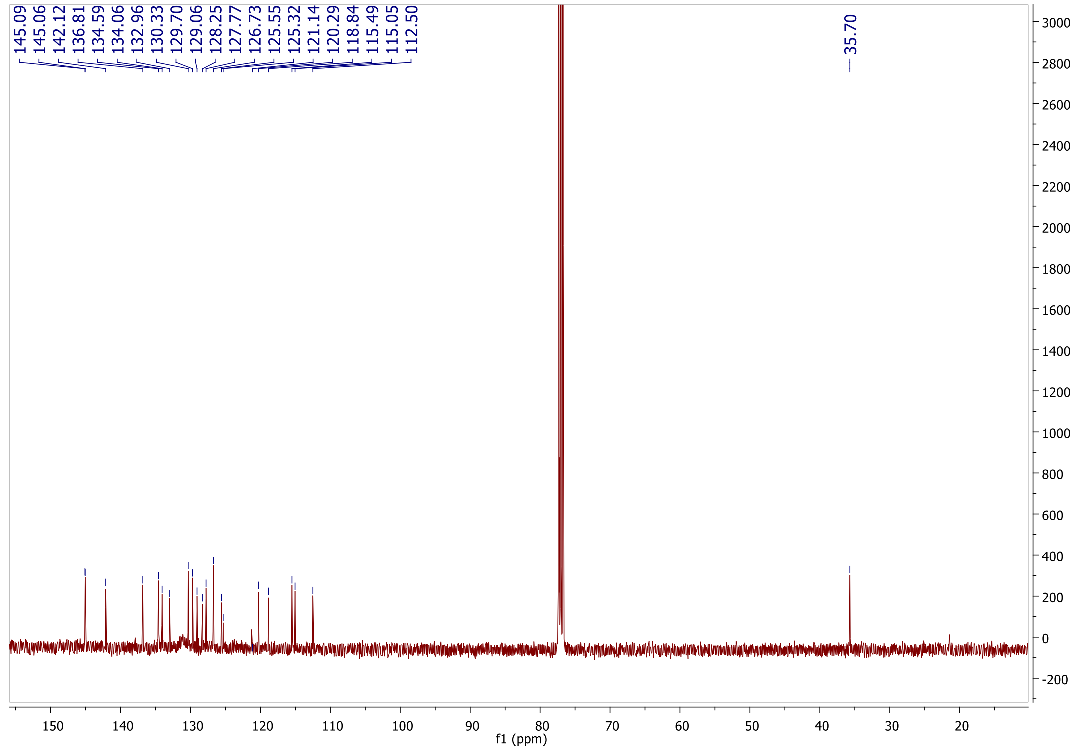

Figure S2. ^{13}C-NMR 100 MHz spectrum of compound **3a** ($CDCl_3$)

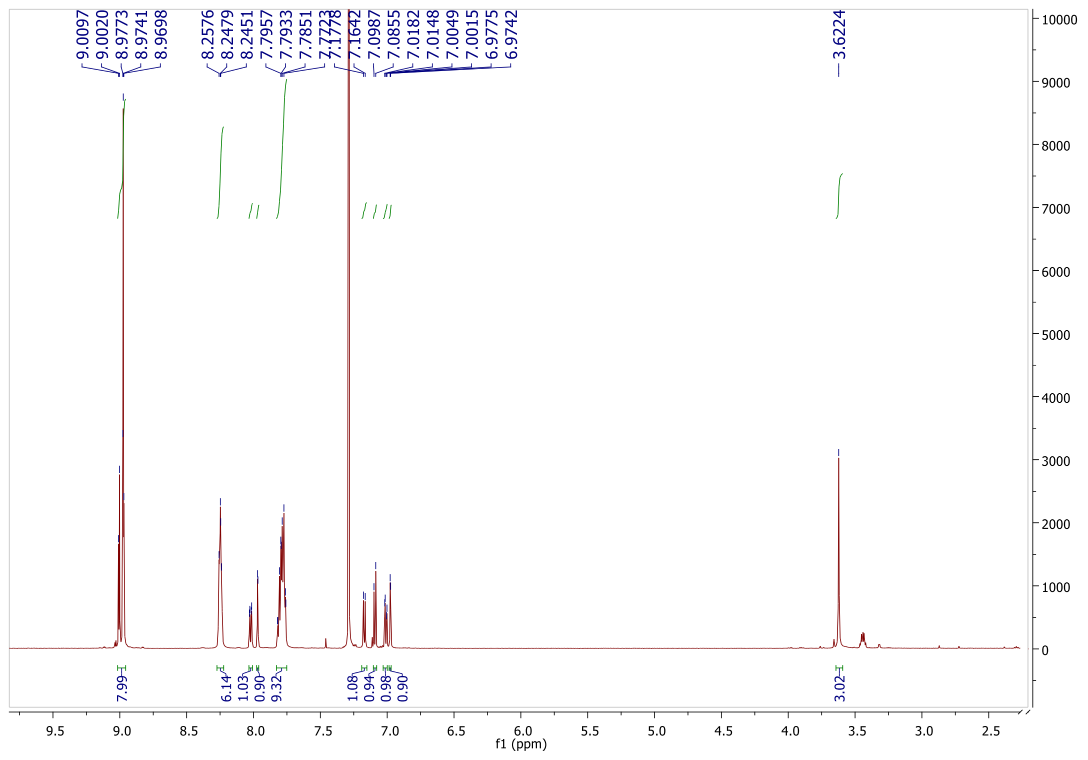

Figure S3. ^{1}H-NMR 600 MHz spectrum of **5a** (CDCl3)

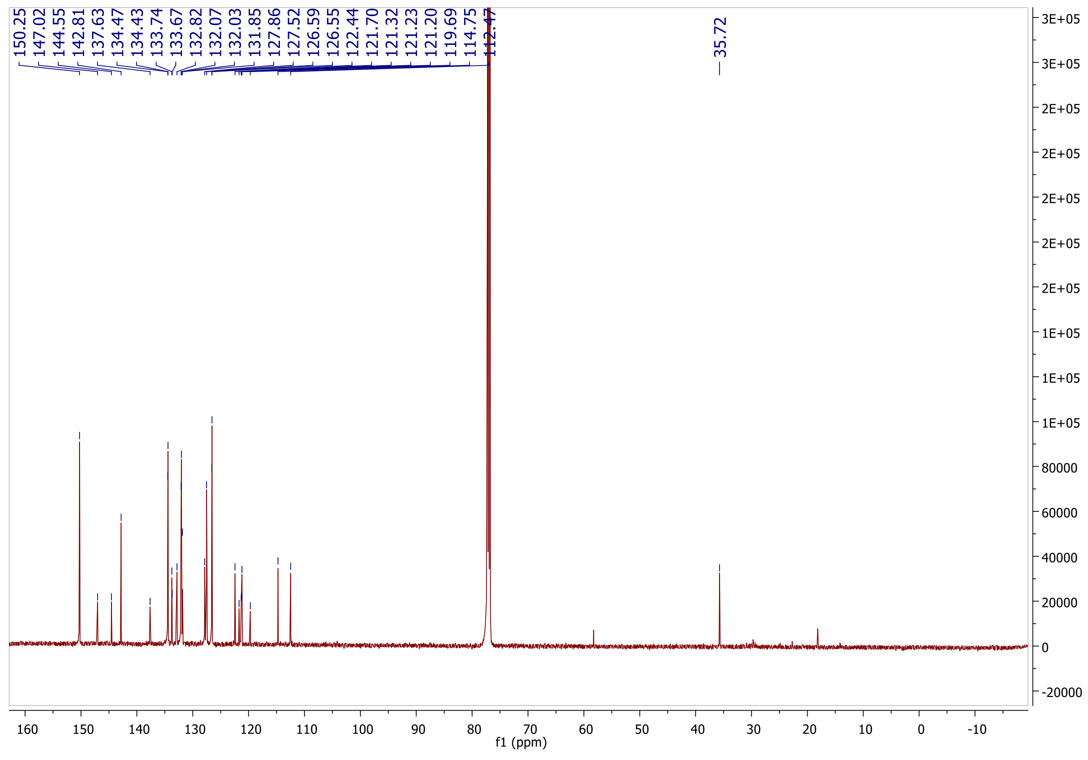

Figure S4.^{13}C-NMR 150 MHz spectrum of **5a** (CDCl3)

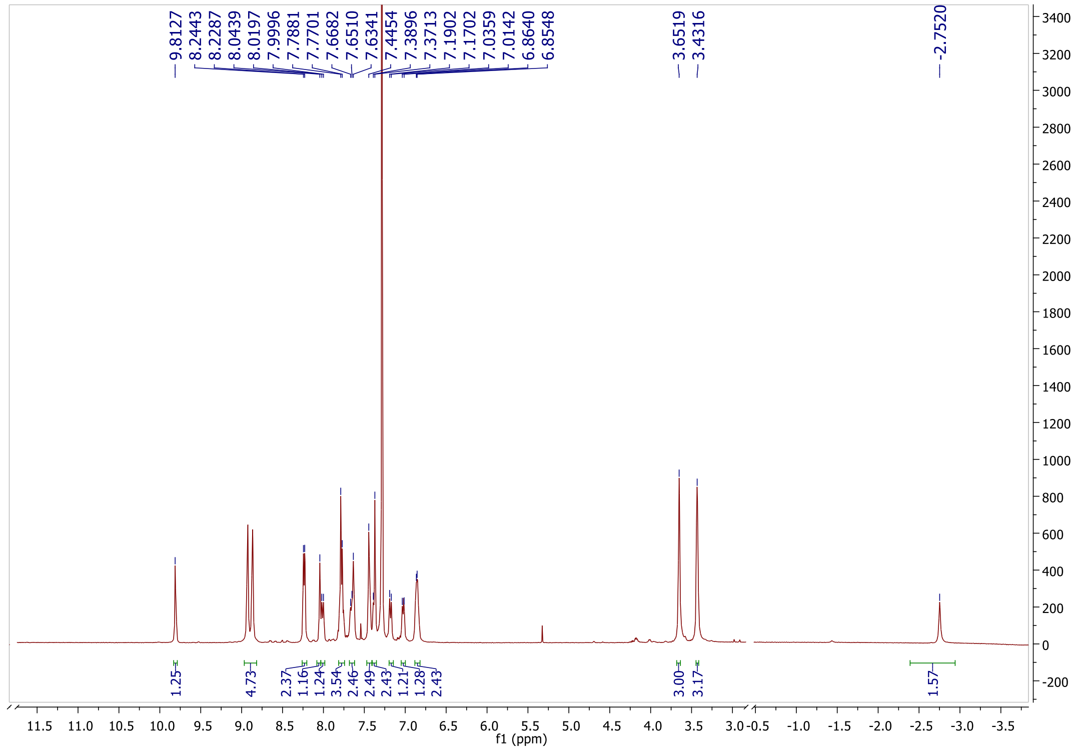

Figure S5.^{1}H-NMR 400 MHz spectrum of compound **13** ($CDCl_3$)

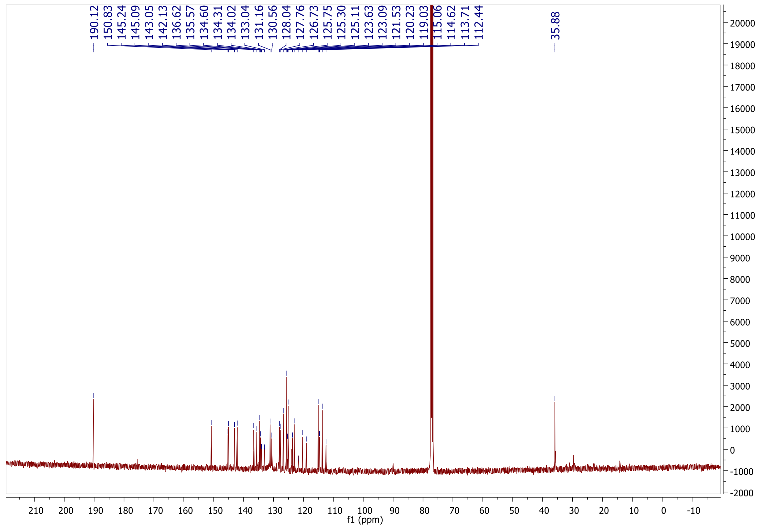

Figure S6.^{13}C-NMR 100 MHz spectrum of compound **13** ($CDCl_3$)

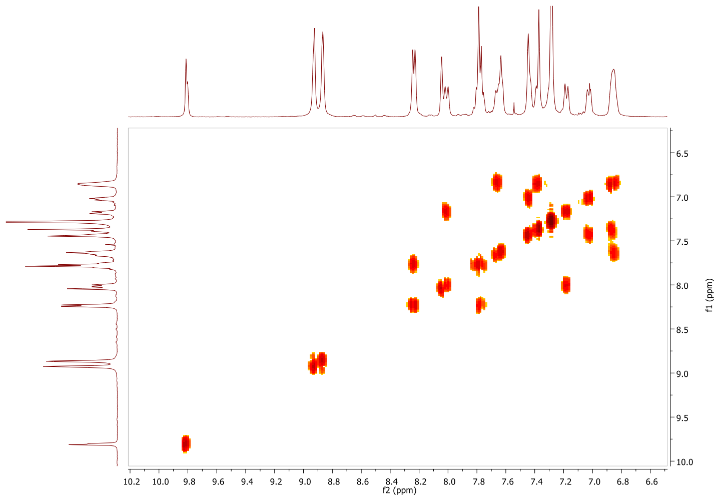

Figure S7. 2D-NMR(^{1}H-^{1}H) COSY 400MHz spectrum of compound **13** (aromatic region, $CDCl_3$)

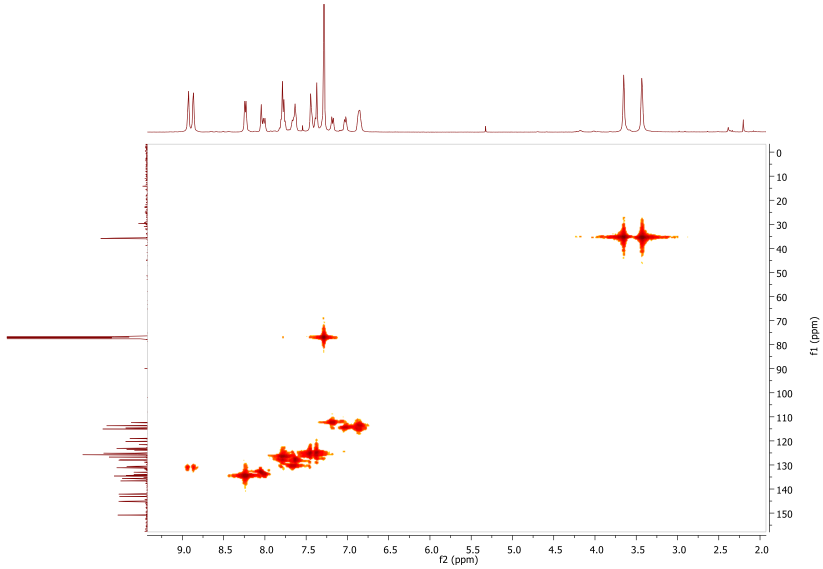

Figure S8. 2D-NMR (^{1}H-^{13}C) HMQC 400MHz spectrum of compound **13** ($CDCl_3$,)

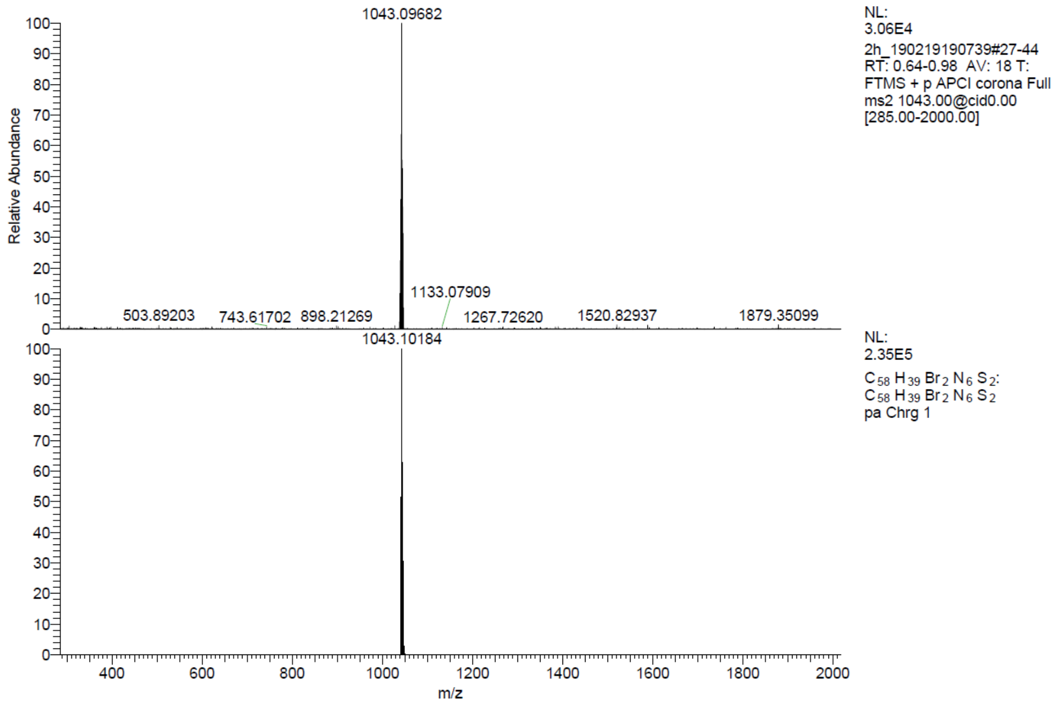

Figure S9. HRMS spectrum of *5,15-diphenyl-10,20-bis(7-bromo-10-methyl-10H-phenothiazin-3-yl)-21,23H-porphyrin* **3a**

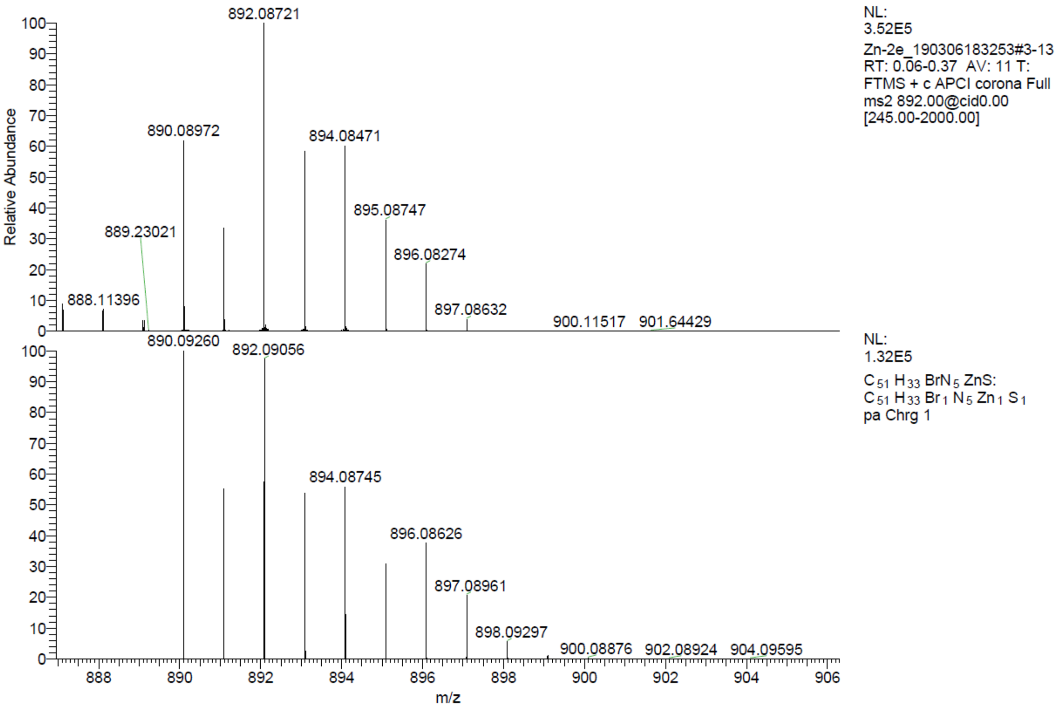

Figure S10. HRMS spectrum of *5,10,15-triphenyl-20-(7-bromo-10-methyl-10H-phenothiazin-3-yl)-21,23-Zn-porphyrin* **4a**

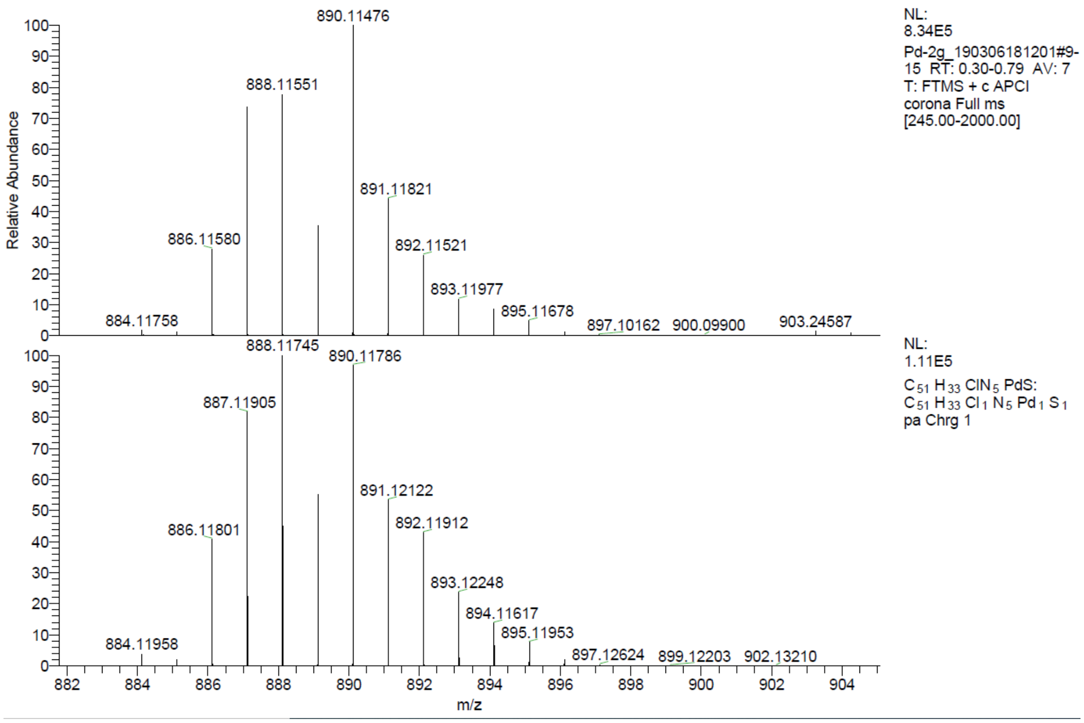

Figure S11. HRMS spectrum of *5,10,15-triphenyl-20-(8-chloro-10-methyl-10H-phenothiazin-3-yl)-21,23-Pd-porphyrin* **5b**

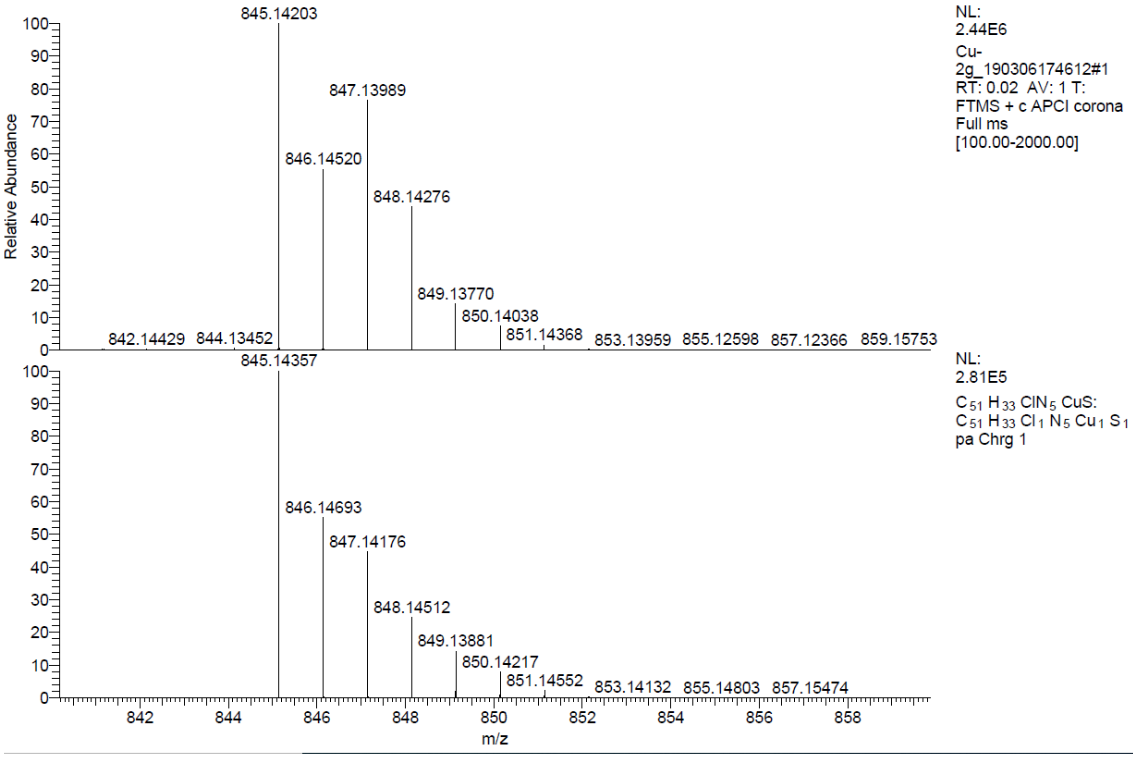

Figure S12. HRMS spectrum of *5,10,15-triphenyl-20-(8-chloro-10-methyl-10H-phenothiazin-3-yl)-21,23-Cu-porphyrin* **5d**

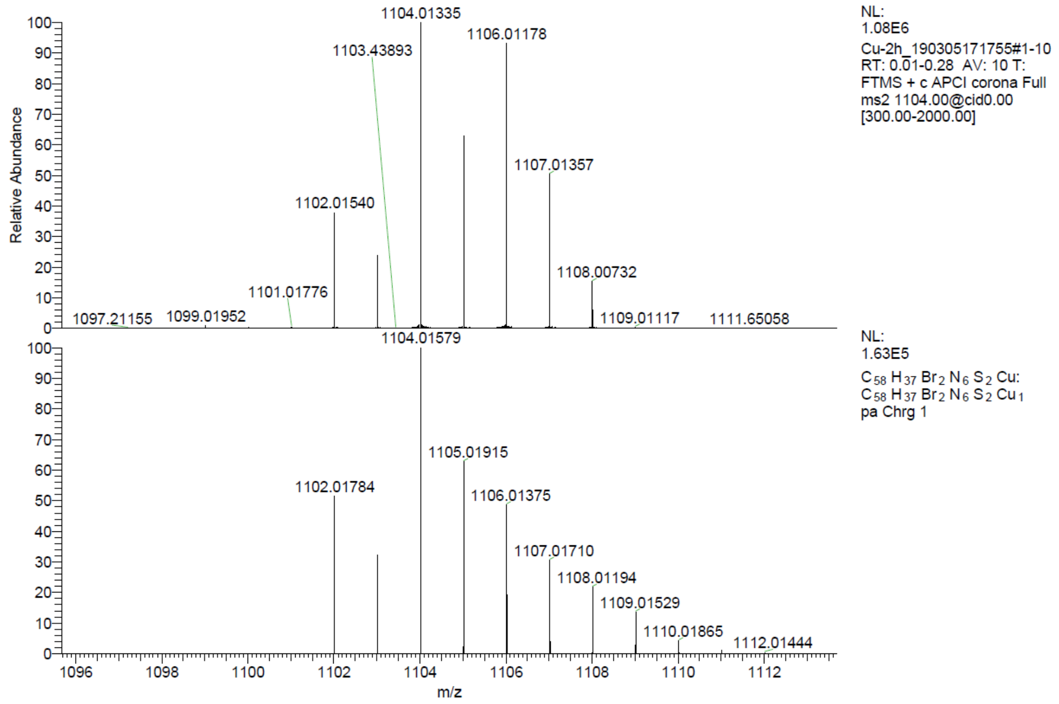

Figure S13. HRMS spectrum of *5,15-diphenyl-10,20-bis(7-bromo-10-methyl-10H-phenothiazin-3-yl)-21,23-Cu-porphyrin* **6d**

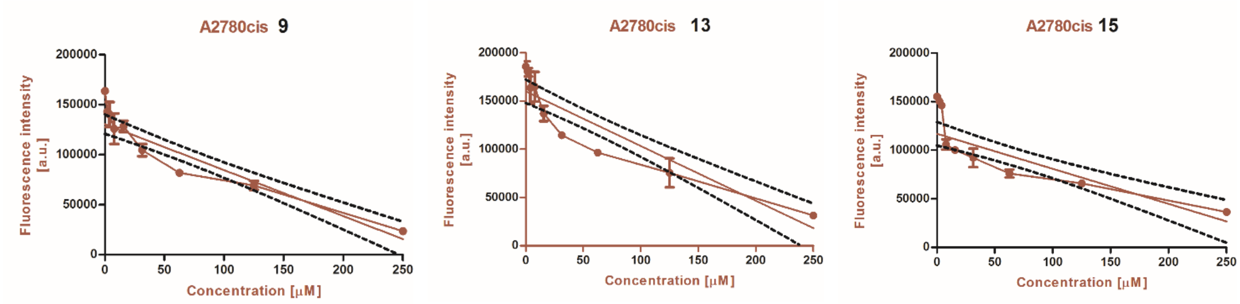

Figure S14. Linear regression of the metabolic rate of ovarian cancer cell line A2780cis *versus* the concentration of the fluorescent dyes: **9, 13, 15.**

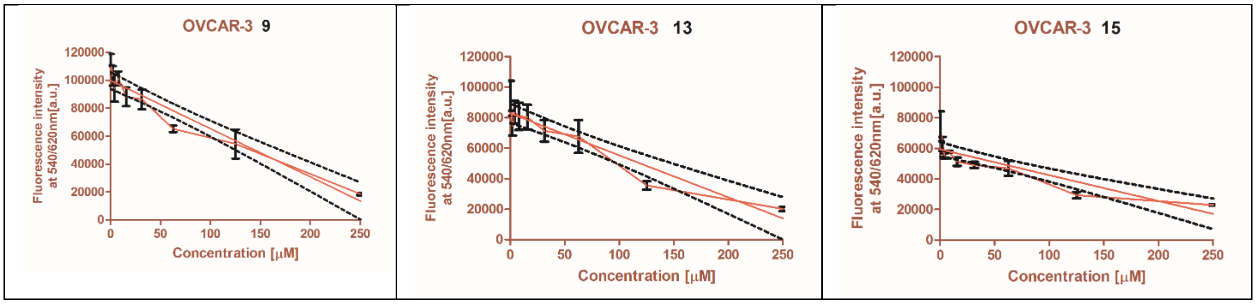

Figure S15. Linear regression of the metabolic rate of ovarian cancer cell line OVCAR-3 *versus* the concentration of the fluorescent dyes: **9, 13, 15.**
